# Nutritional Status Driving Infection by *Trypanosoma cruzi*: Lessons from Experimental Animals

**DOI:** 10.1155/2011/981879

**Published:** 2011-04-14

**Authors:** Guilherme Malafaia, André Talvani

**Affiliations:** ^1^Departamento de Ciências Biológicas, Núcleo de Pesquisas e Estudos Ambientais e Biológicos, Instituto Federal de Educação, Ciência e Tecnologia Goiano—Campus Urutaí, Rodovia Geraldo Silva Nascimento km 2.5, 75790-000 Urutaí, GO, Brazil; ^2^Laboratório de Doença de Chagas, Programa de Pós-Graduação em Ciências Biológicas, Núcleo de Pesquisa em Ciências Biológicas (NUPEB), Universidade Federal de Ouro Preto (UFOP), 35400-000 Ouro Preto, MG, Brazil

## Abstract

This paper reviews the scientific knowledge about protein-energy and micronutrient malnutrition in the context of Chagas disease, especially in experimental models. The search of articles was conducted using the electronic databases of SciELO (Scientific Electronic Library Online), PubMed and MEDLINE published between 1960 and March 2010. It was possible to verify that nutritional deficiencies (protein-energy malnutrition and micronutrient malnutrition) exert a direct effect on the infection by *T. cruzi*. However, little is known about the immunological mechanisms involved in the relationship “nutritional deficiencies and infection by *T. cruzi*”. A hundred years after the discovery of Chagas disease many aspects of this illness still require clarification, including the effects of nutritional deficiencies on immune and pathological mechanisms of *T. cruzi* infection.

## 1. Introduction

The protein-energy malnutrition (PEM) and micronutrient malnutrition (MNM) are common problems worldwide and occur in both developing and developed nations. In the developing world, it is a reflection of socioeconomic, political, or environmental factors, and, in developing countries, PEM usually occurs in the context of chronic diseases [1]. Chagas disease is a chronic parasite illness caused by the hemoflagellate protozoan *Trypanosoma cruzi*, affecting millions of people in developing nations in the South and Central Americas. It is estimated that 15-16 million people are infected with *T. cruzi* in Latin America and 75–90 million are at risk of infection [2]. Due the prevalence of Chagas disease and PEM-MNM together, they are, directly or indirectly, responsible for millions of deaths worldwide [3, 4].

PEM is a major public health problem in the tropical and subtropical regions of the world and often arises during protein and/or energy deficit due to nutritional inadequacy, infections, and poor socioeconomic and environmental conditions [3]. It is the most common nutritional disorder affecting children in developing countries and the third most common clinical disorder of childhood in such countries. PEM has a lasting effect on immune functions, growth and development of children, learning ability, social adjustment, work efficiency, and productivity of labor. It seems that many deaths from PEM occur as a result of outdated clinical practices, and an improving of these practices appears to be essential to reduce this rate of morbidity and mortality [5]. PEM is responsible, directly or indirectly, for 54% of the 10.8 million deaths per year in children under 5 and contributes to every second cause of death (53%) associated with infectious diseases among children under 5 years of age in developing countries [1]. However, PEM is also observed in adults. According to Sawaya [6], adults who have an inadequate food intake may have an increased susceptibility to diseases, including infectious diseases, weight loss, low immunity, damage to the gastrointestinal mucosa, loss of appetite, poor absorption of nutrients, and major changes in metabolism. In addition, adult PEM hospitalized patients are a highly prevalent reality, having as direct consequences the increase of morbidity and mortality, drawn-out hospitalization time, and, therefore, raising the costs for the health system [7–10]. All these consequences provide social, physiological, and psychological damage in adults. 

Another clinical condition that also represents an important issue to the nutritional disorders is the MNM (including vitamins and trace minerals deficiencies). From the public health point of view, MNM is a risk factor for many other acute and chronic diseases and can contribute to high rates of morbidity and even mortality. It has been estimated that micronutrient deficiencies account for about 7.3% of the global burden of disease, with iron and vitamin A deficiency ranking among the 15 leading causes of the global disease burden [11].

According to some authors, there is a close association between PEM/MNM and increased severity of infections by various infectious organisms [12–17], including HIV/AIDS [18], tuberculosis [19], malaria [20], and leishmaniasis [21–23]. Despite this new evidence about nutritional deficiencies and host immune response against protozoan, few studies have been developed about the *T. cruzi* infection. However, the distribution of *T. cruzi* infection overlaps regions with high prevalence of PEM, as we can see, for instance, in the study of De Andrade and Zicker [24].

Thus, the purpose of this paper is to provide an update on what is known about the synergy between Chagas disease and nutritional deficiencies (PMN and MNM), especially in experimental models, and discuss the main studies involving both diseases. Since nutritional deficiency and *T. cruzi* infection are frequent serious problems currently, we wonder whether a one-way dietary intervention might be useful to eliminate PMN and MNM and, indirectly, benefit *T. cruzi*-infected host mammalians under specific treatment or not.

## 2. Methodology

For this study, a bibliographical search was used, including empirical reports, reviews, comments, reports from professional associations, books, editorials, and annals of congresses published in diverse languages. The search of articles was conducted using the electronic databases of SciELO (Scientific Electronic Library Online), PubMed, and MEDLINE published between 1960 and March 2010. For selection of scientific papers, the following criteria were used: (i) papers about PEM, MNM, and Chagas disease, considering the specific aspects on these diseases; (ii) papers about the epidemiology and prevalence of PEM, MNM, and Chagas disease; (iii) studies published in specialized magazines with considerable impact. The following search terms were used on their own and in combination: “Chagas disease,” “*Trypanosoma cruzi*,” “malnutrition,” “protein-energy malnutrition,” “micronutrient malnutrition,” “protein deficiency”; “vitamins deficiency”; “iron deficiency,” “zinc deficiency” and “selenium deficiency”. All retrieved studies were evaluated by reading the abstracts; and full papers were retrieved for articles that could not be rejected based on abstract alone. References from the retrieved papers were also manually searched for additional relevant studies. 

Descriptive studies were considered, those that described the epidemiology, prevalence, and pathology of Chagas disease and/or PEM/MNM. The studies on determination were considered, those that explored, directly or indirectly, the relationship between the PEM, MNM, and Chagas disease. Furthermore, studies that described the consequences of PEM and MNM on the immune system were also consulted to base the discussion.

## 3. Results and Discussion

### 3.1. Epidemiology, *T. cruzi* Infection, and Clinical Evolution: General Aspects

 As revised by Coura and Dias [2], Chagas disease originated millions of years ago as an enzootic infection of wild animals and began to be transmitted to humans as an anthropozoonosis when man invaded wild ecotopes. While evidence of human infection has been found in mummies up to 9,000 years old, endemic Chagas disease became established as a zoonosis only in the last 200–300 years, as triatomines adapted to domestic environments [25]. The geographical distribution of Chagas disease, including its reservoirs and its vectors, extends from the Southern United States to Southern Argentina and Chile. Thus, it covers all Americas, and 90 million people in this region are exposed to infection. It is currently estimated that 15 million people present *T. cruzi* infection or carry the disease [2]. In Brazil, for example, recent data from the Brazilian Ministry of Health confirmed the occurrence of 2,476 cases (1,603 vectorial, 16 transfusional, and 7 transplacentary transmission, as well as 850 cases of nonidentified form of transmission) of acute Chagas disease from 2001 to 2006 with 95 deaths. Furthermore, recently the transmission of Chagas disease by oral route has gained evidence through the consumption of Açaí, the fruit of a palm of the family Aracaceae, especially among Amazonian population that consumes açaí juice daily. It is believed that the contamination occurs when inadvertently triatomines are crushed during processing releasing infective forms of parasites stationed in their stools. Besides, consumption of raw meat from infected mammalian sylvatic hosts is also described for Chagas oral transmission [26]. 

There is a general consensus that during chronic *T. cruzi* infection, the host immune system induces complex processes to ensure the control of parasite growth while preserving the potential to mount and maintain a life-long controlled humoral and cellular immune response against the invading pathogen. Recently, increasing focus on innate immunity suggests that chronic *T. cruzi* infection may cause morbidity when innate effectors functions or the downregulation of adaptive regulatory mechanisms are lacking [27]. In this context, stable asymptomatic host-parasite interactions seem to be influenced by the effector/regulatory balance with the participation of macrophages, natural killer (NK) and CD8^+^ T cells in parallel with the establishment of regulatory mechanisms mediated by natural killer T (NKT) and regulatory T (Treg) cells. Moreover, a balanced innate immune activation state, apart from Treg cells, may play a role in controlling the adverse events triggered by the massive antigen release induced by trypanosomicidal agents during Chagas disease etiological treatment. 

The determinants of Chagas disease come from the quantity of parasites in the initial infection (number of trypomastigotes), the lineage of the inoculated *T. cruzi* (I, II, Z3, or hybrid I/Z3, today reclassified as TcI–TcVI), the reinfections, the quality of the strains and clones (biodemes); the specific clonal-histotrophic receptors, and the mammalian host immune response [28–32].

After *T. cruzi* infection, this disease presents two successive phases: acute and chronic. Acute infection is often asymptomatic or may manifest as a self-limited febrile illness that lasts 4–8 weeks. Clinical manifestations of chronic Chagas disease are related to the pathologic involvement of the heart, esophagus, and/or colon [32, 33]. Parasites play a fundamental role in the genesis and development of organs lesions by sequentially inducing an inflammatory response, cellular and tissue lesions, and fibrosis [34].

Complexities of the pathology of Chagas disease and the diversity of its clinical manifestations have made the understanding of its pathogenesis difficult. However, the inflammatory background (cellular, humoral, and/or autoimmune responses) concerning this disease has been largely investigated, especially in cardiac tissue. The infiltrate in chronic Chagas heart disease consists of lymphocytes, and to a lesser extent, macrophages, eosinophils, plasma cells, neutrophils, and mast cells. T cells predominate and CD8^+^ lymphocytes are two to three times more abundant than CD4^+^ cells [35]. The most part of this leukocyte recruitment into the heart tissue is driven by chemokine and chemokine receptors and, their overexpression is clearly associated with heart dysfunction [36–38]. The perpetuation of leukocyte in parasites is rarely found in the heart but parasite DNA can be detected in some inflammatory lesions. Myocyte destruction is followed by dissolution of the inflammatory cell infiltrate and replacement with connective tissue. This fibrosis is composed by collagen types I and III and, over the years, is thought to be responsible for decreased contractility of the heart, diminished cardiac muscle mass, and destruction of the intrinsic innervations. Lesions similar to those in the myocardium are found in both the epicardium and the endocardium.

### 3.2. Evidences of Relation between Nutritional Deficiencies and *T. cruzi* Infection

The first critical report describing malnutrition and infectious disease related to mortality and morbidity was probably by Scrimshaw et al. [39]. This topic suggests a vicious cycle involved, in which PEM/MNM increases the susceptibility to infections [16]. In response to infection, the immune system first executes innate defense functions and then subsequently acquired host defense functions of great diversity. Both processes involve activation of immune cells and synthesis of an array of molecules requiring DNA replication, RNA expression, and protein synthesis, and secretion and therefore consume additional anabolic energy. Mediators of inflammation further increase the catabolic response. Consequently, the nutritional status of the host critically determines the outcome of infection. Almost all nutrients in the diet play a crucial role in maintaining an “optimal” immune response; then deficiency can have negative consequences on immune status [40] and susceptibility to a variety of pathogens [41], including *T. cruzi*.

### 3.3. Protein Deficiency and Infection by *T. cruzi*

Regarding protein deficiency and *T. cruzi* infection, only the studies of Machado et al. [42], Gomes et al. [43], Carlomagno et al. [44], and Cintra et al. [45] (involving experimental models), and De Andrade and Zicker [24] (involving human) were found. Machado et al. [42] studied the relation between the cardiac noradrenalin and the PEM in chronic experimental Chagas disease in rats (in this case, no immune interaction was evaluated). The results indicate that protein deficiency at least delays the recovery of noradrenalin levels that normally occurs during the chronic phase of experimental Chagas disease. 

The study of Gomes et al. [43] was conducted to determine the effects of severe protein restriction (4.75, 9.5, 14.25 and 19% of protein in isocaloric diets with normal content of mineral and vitamins) on parasitemia and mortality of mice acutely infected with Y and CL strains of *T. cruzi*. Only severe protein restriction (4.75%) induced decrease in resistance to the infection with both Y and CL strains of *T. cruzi*, which resulted in higher parasitemia and mortality. The inflammatory lesions in heart and skeleton muscle were less extensive in groups with severe protein restriction despite the increased number of parasite in muscle cells. According to authors, depression of immune mechanisms could be responsible for the reduced resistance and inflammatory reaction after *T. cruzi* infection in highly protein-restricted animals. However, authors did not perform any evaluation of immune response or inflammatory response to prove these evidences. In this case, considering that the micronutrients and macronutrients favor an adequate immune response and that they are necessary for cell proliferation and production of antibodies and other immune proteins, it is questionable whether malnourished patients can regulate their immune response and, thus, would not be harmed by inflammatory response. There are no studies concerning these aspects.

 In order to determine if dietary proteins are responsible for alterations in the course of *T. cruzi* infection in calorie depleted mice, Carlomagno et al. [44] measured the effects of diets with reduced protein contents in the same animal model. BALB/c mice receiving 20% (NP) or 2% (LP) protein diets were injected with 100 blood trypomastigotes 15 days after diet starts. Onset of parasitemia was detected earlier in the LP group, but on day 13th post infection (pi), parasitemia levels, as well as survival time, were not statistically different from NP animals. Although a chronic state could not be reached with animals infected with 15 blood trypomastigotes and receiving a 6% (SP) protein diet, those animals sacrificed at 30 days pi showed statistically different DTH indexes, but not different specific antibody titers, compared to NP animals. To determine the effect of the moderate protein deficiency upon the degree of protection conferred by a flagellar fraction (FF) of *T. cruzi* which protects well-nourished animals against a lethal challenge of the parasite, SP and NP mice immunized with 5 weekly doses of FF were challenged with 1000 metacyclic trypomastigotes. Blood parasite levels were higher for vaccinated SP animals compared to NP controls. Survival was also significantly lower for animals receiving the low protein diet. 

Cintra et al. [45] have studied the influence of dietary protein on *T. cruzi* infection in germ-free (GF) and conventional (CV) mice. To do so, GF and CV mice were fed on diets containing 4.4, 13.2, or 26.4% of protein. The protein deficiency affected less the GF than the CV mice regarding weight gain, hemoglobin, plasma protein and albumin levels and water and protein contents of the carcass. However, in the presence of *T. cruzi*, GF mice were more affected by this dietary, reducing hemoglobin levels, red blood cell count, and water and protein contents in the carcass.

In a human study, De Andrade and Zicker [24] examined the possible association between *T. cruzi* infection and chronic PEM in Brazilian children. In a cross-sectional survey conducted in the 1900s involving 153 children 7–12 years old from 60 village schools in central Brazil presenting positive serology to Chagas disease, seropositive children had a 2.4-fold risk (95% CI 1.4–4.0) of being stunted (z-score < 2.0 of height-for-age) when compared to uninfected children even after adjusting for confounding variables. Being underweight (z-score < −2.0 of weight-for-age) was also statistically associated with seropositivity to *T. cruzi* (OR = 2.8, 95% CI 1.4–5.6). Results of this study suggested that further studies on nutrition and metabolism are required to look into a possible physiopathological mechanism for this association. Moreover, with these studies we can venture the following questions: would the protein deficiency be, in part, responsible for the worsening of Chagas disease? Or may *T. cruzi* infection lead hosts to nutritional deficiency? Changes in the digestive tract are described in individuals with Chagas disease [46]. In these cases, such changes may lead patients to nutritional deficiencies, changing the digestion and/or absorption of micronutrients and macronutrients. After more than 10 years of publication of the paper by De Andrade and Zicker [24], little is still known about this subject.

Despite few studies mentioning aspects concerning “Chagas disease and protein deficiency,” there is no doubt that protein deficiency affects general immunological aspects, which together may interfere in those malnourished Chagas disease patients affecting “host-parasite” interactions. Regarding innate immunity, there are multiple and interconnected mechanisms interfering in microbial infections such as phagocytosis, NK cells, complement-mediated lysis, and opsonization. Anstead et al. [21], studying the effect of the malnutrition on innate immunity and early visceralization following *Leishmania donovani* infection, observed that malnutrition caused a failure of lymph node barrier function after *L. donovani* infection, which may be related to excessive production of prostaglandin E_2_ (PGE_2_) and decreased levels of IL-10 and nitric oxide (NO). In the occasion, a murine model of polynutrient deficiency (diets deficient in iron, zinc, and calories) demonstrated that malnourished mice had altered innate immune defense indicating an increased risk of visceralization following *L. donovani* infection.

Later, the study of Anstead et al. [47] demonstrated that, after stimulation with IFN-*γ*/LPS, macrophages from malnourished mice, as compared with their well-nourished control mice counterparts, produced less TNF-*α*, IL-10, and NO. According to the authors, the deficits in TNF-*α* expression and low production of NO have significant consequences in host defense. NO, for instance, is an essential regulatory molecule in host protective responses, acting as a potential host-destructive mediator in severe pathologies, mainly in infectious diseases. Besides, the low levels of IL-10 produced by the macrophages from the malnourished mice may have adverse effects on the innate immune response [47]. 

Abe et al. [48] demonstrated that, in mice with experimental PEM, phagocytosis and production of reactive oxygen intermediates (ROIs) and reactive nitrogen intermediates (RNIs) released by macrophages were diminished, as well as the function of antigen presentation to T cells by dendritic cells. PEM in mice, challenged by experimental peritonitis, resulted in impaired immune cell migration and extravasation as indicated by reduced numbers of CD11b/CD18^+^ cells at the site of infection, involving lower concentrations of the chemokine MIP-2 or CXCL2.

In humans, there is a close-to-normal neutrophil chemotaxis and phagocytosis and minor defects in the generation of ROIs and bacterial killing in malnourished patients [49]. A significant depression of serum opsonin activity [50] explains these changes. All components of the complement system—except C4—have been observed to be depressed in malnourished patients, particularly C3 and factor B [51].

The NK cells are considered particularly important in viral diseases, but they may also be important in resistance to some prokaryotic intracellular parasites, such as *T. cruzi* [52–54]. Early findings of depressed NK cell lytic activity in blood mononuclear cells of children with PEM have been extended from a study of protein-deficient weanling mice [55]. Surface marker analysis revealed that the low NK cell lytic activity of splenic mononuclear cell suspensions resulted from a depression in lytic cellular activity.

Regarding adaptive defenses, the mechanisms of action resulting from protein deficiency on this system are also multiple. Keusch et al. [56] reported that, in malnourished children, there was a significant decrease in T-cell function and an increase in null cells that apparently failed to further differentiate. The structure and function of the thymus were damaged, and T-cell memory response to antigens was reduced [57]. 

 According to Woodward [58], lymphoid involution, as indicated by the size and cellularity of secondary lymphoid organs, is a characteristic of PEM, and according to its degree there is a depression in adaptive immunocompetence. At the same time, the recirculating (i.e., surveillance) pool of lymphocytes in a weanling mouse model of wasting protein deficiency indicates that measures of lymphoid organ size may yield an inflated impression of the extent of lymphoid involution in PEM [58]. 

Imbalance among critical subsets of lymphocytes may also contribute to the initiation or the continuation of PEM-induced immunodepression. PEM leads to long-lasting immune defects characterized by leucopenia, decrease of CD4/CD8 ratio and increase number of CD4/CD8 double-negative T cells, and, therefore, the appearance of immature T cells in the periphery [59]. Circulating T lymphocytes from infected children with PEM had lower expression of the activation marker CD69 and predominantly showed an intermediate (CD45RA^low^/CD45RO^low^) rather than a memory phenotype (CD45RO^high^) when compared to healthy donors [60, 61]. These T cells were biased towards type 2 T helper cell (Th2) responses, represented by decreased IFN-*γ*/IL-2 (T helper cell—type 1/Th1) phenotype and increased IL-4/IL-10 (type-2—Th2) production [62]. Experimentally undernourished weanling mice had predominantly T cells of the naïve quiescent phenotype (CD45RA^+^/CD62L^+^) [63, 64], where IFN-*γ* responses were depressed and IL-10 and the Th2-associated antibody, IgE, were increased, while IL-4 production remained normal [65]. 

Antibody production in malnourished individuals seems to be depending on the types of antigens involved. Malnourished patients appear to respond well to tetanus [66] and flagellar antigens but not to the polysaccharide antigens [67], which suggests that there is a defect in response to carbohydrates. Reduced antibody responses to polysaccharide antigens of encapsulated bacteria such as *Streptococcus pneumoniae* and *Haemophilus influenzae* exacerbate susceptibility to these pathogens [68].

### 3.4. Vitamin Deficiency and Infection by *T. cruzi*

#### 3.4.1. Vitamin or Complex B

Vitamins are essential constituents of our diet that have long been known to influence the immune system [69]. Concerning the studies conducted on the theme “vitamins deficiency and *T. cruzi* or Chagas's disease,” the pioneering study of Yaeger and Miller [70] was conducted to determine the effect of thiamine (vitamin B_1_) deficiency on *T. cruzi* infections in albino rats. The following conclusions were drawn from vitamin B_1_-deficient rats: (i) parasitemia were generally higher than in control groups and (ii) cardiac lesions were more extensive and tissue parasites more common, suggesting once more that the presence of the etiologic agent in concomitance with the nutritional deficiency status changes completely the course of the disease.

However, few studies exist about effects of vitamin B_1_ deficiency on immune system, which could explain the susceptibility of vitamin B_1_-deficient rats to *T. cruzi* infection. It is known that the active form of thiamine is essential for its coenzyme functions in carbohydrate and branched chain amino acids metabolisms. The thiamine pyrophosphate is essential in decomposition reactions of glucose in energy. Albino rats maintained for 13 weeks on a riboflavin- (vitamin B_12_) deficient diet did not exhibit a significant increase in susceptibility to *T. cruzi* infection. The slight increase in severity of infection as evidenced by cardiac damage and higher parasitemia was attributed to inanition rather than to deficiency of the vitamin [71]. On the other hand, when pantothenate (vitamin B_5_) deficiency was used on the *T. cruzi* infection, also in albino rats, it was observed that (i) *T. cruzi* produced more severe infections in vitamin B_5_-deficient rats than in normal ones and inanition control groups of rats (ii) parasitemia was higher and cardiac lesions were usually more extensive in the vitamin B_5_-deficient animals, and, finally, (iii) the incidence of lung infections among vitamin B_5_-deficient rats suggests that this deficiency may increase the susceptibility of rats to spontaneous respiratory disease [72]. This same investigative group also demonstrated that rats maintained on a diet from pyridoxine (vitamin B_6_) and vitamin A, were more susceptible to *T. cruzi* infection, with higher parasitemia and myocarditis [73, 74]. 

Early stages of vitamin B_1_ deficiency may be accompanied by nonspecific symptoms that may be overlooked or easily misinterpreted [75]. The clinical signs of deficiency, in human, include anorexia, weight loss, mental changes such as apathy, decrease in short-term memory, confusion, and irritability, muscle weakness, and cardiovascular effects such as an enlarged heart [76–79]. The central nervous system damage induced by vitamin B_1_ deficiency seems to be simultaneously brought by (i) an increase in free radical production, (ii) oxidative stress, (iii) axonal membrane damage, (iv) disturbed myelinogenesis, (v) selective neuronal death, and (vi) glutamatemediated excitotoxicity [80–82]. Vitamin B_1_ deficiency also severely affects the peripheral nervous system [83].

Equally important is the role of vitamin B_6_ in the immune functions. As revised by Rall and Meydani [84], animal and human studies suggest that vitamin B_6_ deficiency affects both humoral and cell-mediated immune responses. Lymphocyte differentiation and maturation are altered by deficiency, delayed-type hypersensitivity responses are reduced, and antibody production may be indirectly impaired. In addition, deficiency of the vitamin has been associated with immunological changes observed in the elderly, persons infected with human immunodeficiency virus (HIV), and those with uremia or rheumatoid arthritis.

#### 3.4.2. Vitamin A

It is known that the vitamin A deficiency impairs innate immunity [85], which is important for the control of Chagas disease [27]. According to Stephensen [85], the vitamin A deficiency impairs innate immunity by impeding normal regeneration of mucosal barriers damaged by infection, as well as by diminishing the function of neutrophils, macrophages, and natural killer cells. Vitamin A is also required for adaptive immunity and plays a role in the development of both T helper (Th) and B cells. In particular, vitamin A deficiency diminishes antibody-mediated responses directed by Th2 cells, although some aspects of Th1-mediated immunity are also diminished. In addition, Mucida et al. [86] have recently shown that the vitamin A metabolites, including retinoic acid (RA) and ligands for retinoic acid-related nuclear receptors, play pleiotropic roles in various biological processes. Authors described that RA functions as a key modulator of transforming growth factor-beta- (TGF-beta-) driven immune deviation, capable of suppressing the differentiation of interleukin-17 secreting T helper cells (T(H)17) and conversely promoting the generation of Foxp3(+) T regulatory (Treg) cells.

#### 3.4.3. Vitamin E

More recently, another study with vitamin E deficiency was shown in experimental acute phase of Chagas disease [87]. Using rats, authors observed that vitamin E deficiency induced the decline in CD45RA^+^CD3^−^ B cells and CD3^+^CD4^+^ T lymphocytes in the peripheral blood and an exacerbation of myocarditis and sympathetic denervation of ventricles [87]. These results are in agreement with data from Moriguchi et al. [88], demonstrating that vitamin E deficiency results in low differentiation of T lymphocytes in the thymus, mainly in CD4^+^ T cells. The essential role of these two cellular populations in resistance to *T. cruzi* infection is well known from the literature [89, 90]. During *T. cruzi* acute infection, the antibody response mounted by the host, which is essential to be strictly dependent on the help provided by (or on the activation of) CD4^+^ T-lymphocytes activation, is decisive for the outcome of the infection [91]. Carvalho et al. [87] also observed that vitamin E deficiency induced monocytosis, as well as an increased differentiation rate of monocytes to macrophages, as revealed by immunohistochemistry. In summary, these observations point to an increased tissue parasitism in vitamin E-deficient rats. This may suggest that vitamin E deficiency induces an exacerbation of inflammatory status during *T. cruzi* infection and an enhancing of components of the inflammatory response, for example, the secretion of proinflammatory cytokines by macrophages [92–96].

### 3.5. Iron or Zinc Deficiency and *T. cruzi* Infection

Regarding the immune system, several studies have pointed to the importance of iron, mainly because they find that iron deficiency leads to defects in both adaptive and innate response, causing reduction in the phagocytosis by neutrophils, in the activation of T cells and, also, inducing significant changes in the cytokines production [97–99].

Lalonde and Holbein [100] were the first to report the influence of iron status on pathogenicity of *T. cruzi* in C57BL/6 and C3H mice using a promoter of iron deficiency—the iron chelator desferrioxamine. These authors demonstrated that a reduction of intracellular iron stores reduced the pathogenicity of *T. cruzi* infection, both in the moderately resistant C57Bl/6 mouse strain and in the highly susceptible C3H strain.

Pedrosa et al. studied the effect of iron deficiency and iron overload on the evolution of Chagas disease induced by CL, Y, and YuYu strains of *T. cruzi* in CFW or gnotobiotic mice (GN-CFW). The parasitemia was more intense in iron-dextran-treated, mice and *T. cruzi*-specific IgG and IgM antibody levels were raised in the GN group but not in the conventional group [101, 102]. This study suggests that the effects of iron deficiency on *T. cruzi* infection are dependent on host background and *T. cruzi strains*, reinforcing the importance of the genetic diversity of parasites on the course of infection, as our group and others have previously demonstrated [103, 104]. 

More recently, a prolonged treatment with desferrioxamine was tested in experimental model of Chagas disease, in association or not with the anti-*T.cruzi* drug, Benznidazole. It was observed that a decrease of iron in the host leads to *T. cruzi* infection attenuation using hemoculture, ELISA or PCR [105], and iron status of the host influenced the efficacy of therapy with Benznidazole and improved the percentage of infected mice survival [106]. This currently available nitroimidazole drug, Benznidazole, developed empirically over three decades ago, is unsatisfactory due to frequent toxic side effects and limited efficacy, particularly during chronic form of the disease, but this association with a multispectrum of new drugs or diet constituents might improve its use in a low dose with high affectivity against *T. cruzi*. 

Regarding effects of zinc deficiency, several studies have demonstrated the importance of this constituent in the immune system and in the relationship “parasite/host” [107, 108]. In zinc deficiency, thymic atrophy, lymphopenia, decreased mitosis, and decreased serum antibodies [109–111] may occur. In addition, damage can occur in the mucosal barrier of the gastrointestinal tract and lungs, increasing susceptibility to fungi, viruses and bacteria infections [109, 112].

Studies on *T. cruzi* infection have demonstrated the effects of zinc supplementation on the response during the course of experimental disease [113–115]. These studies point in the direction that zinc supplementation enhanced thymocyte and splenocyte proliferation, enhanced production of IL-12 during the acute phase of infection [113], and decreased parasitemia load associated with high levels of IFN-gamma and NO [114, 115]. Brazão et al. [116] have demonstrated that zinc, steroid hormone dehydroepiandrosterone (DHEA), or zinc and DHEA supplementation enhanced the immune response, as evidenced by a significant reduction in parasitemia levels during the acute phase of infection in experimental model. Zinc and DHEA supplementation exerted additive effects on the immune response by elevation of the number of macrophage and by increasing concentrations of IFN-gamma and NO. In addition, Gonçalves-Neto et al. [117] investigated the effects of zinc sulphate (ZnSO_4_) supplementation during pregnancy in mice. For placental parasitism, zinc-supplied group displayed a significant decrease in amastigote nests, suggesting that zinc was partially effective in upregulating host immune response against parasite, probably attenuating the infection in fetuses. Together, these studies suggest that zinc supplementation leads to upmodulating the host immune response and contributing to the reduction of blood parasites and the harmful pathogenic effects of the experimental Chagas disease.

### 3.6. Selenium Deficiency and *T. cruzi* Infection

Selenium is an essential micronutrient for organisms ranging from bacteria to humans. Acting as an antioxidant at the cellular level, this diet constituent provides protection against free radical damage and oxidative stress [118–120]. According to Nève [121], selenium deficiency has been implicated in some cases of congestive cardiomyopathy and other cardiovascular complications, including myocardial infarction. Regarding the study of the effects of selenium deficiency on the *T. cruzi* infection or its consequence (e.g., Chagas cardiomyopathy), Rivera et al. [122] investigated whether selenium status could be involved as a risk factor for the clinical severity of Chagas heart disease. This study involved 170 patients clinically stratified into groups as follows: indeterminate or asymptomatic (IND), cardiac asymptomatic (CARDa); cardiac symptomatic with moderate-to-severe heart dysfunction (CARDb), and healthy adults (HA). Selenium was significantly lower in CARDb than in HA, IND, or CARDa patients, and it presented a positive and significant correlation with ventricular ejection fraction. The decrease in selenium in chagasic patients seems to be a potential biological marker for *T. cruzi* infection and related to the progression of pathology. 

Interestingly, selenium deficiency was investigated in mice experimentally infected with *T. cruzi* during the development of the acute myocarditis [123, 124]. Survival rate was significantly lower in selenium deficient than in control mice, and, at histological evaluation, the severity of myositis was always more intense in the selenium-deficient mice concluding that this deficiency condition, in murine model, is able to increase the severity of *T. cruzi*-induced myositis and mortality of the animals. 

De Souza et al. [125] demonstrated that, although the selenium supplementation does not lead to a general protection during infection, it may help protect the heart from inflammatory damage. More recently, De Souza et al. [126], employing magnetic resonance imaging to noninvasively monitor the effect of selenium supplementation on alterations in the gastrointestinal tract of *T. cruzi*-infected mice, suggested that selenium may be used to modulate the inflammatory, immunological, and/or antioxidant responses involved in intestinal disturbances caused by *T. cruzi* infection. In addition, De Souza et al. [127] also showed that selenium supplementation prevents right ventricular chamber increase and thus can be proposed as a potential adjuvant therapy for cardiac alterations already established by *T. cruzi* infection. 

## 4. Concluding Remarks

In this paper, we discussed about few evidences of nutritional deficiencies (PEM and MNM) and their effects on *T. cruzi* infection. In this way, protein deficiency was shown to delay recovery of noradrenaline levels that normally occurs during chronic phase of experimental Chagas disease. Besides, this protein deficiency also decreases resistance to infection and fails to control parasite replication. Deficiencies in iron and vitamins A, E, B_1_, B_5_, and B_6_ also exacerbated *T. cruzi* infection in experimental models showing correlations between severity and progression of the disease. 

Regarding nutrient supplementation, encouraging results point to the benefits of vitamin E, zinc, and selenium supplementation in cases of experimental *T. cruzi* infection. In this respect, it is important to emphasize that the choice of experimental model is an important task to investigate nutritional deficiencies and Chagas disease, in concomitance or not. Although several animal species have been used (dogs, mice/rats, monkeys and rabbits), there is still a lack of consistency in experimental studies and diversity of methodological. 

Despite these limitations, especially those related to the weak knowledge about mechanisms that govern the relationship between “nutritional deficiencies” and “Chagas disease,” some issues should be conducted to the encouragement of new studies: do PEM and MNM affect the epidemiology and distribution of Chagas disease? Do PEM and MNM affect the diagnosis, pathogenesis, and treatment of this zoonosis? The understanding of immune mechanisms involved in concomitance of PEM/MNM and Chagas disease may conduct new prophylactic and therapeutic strategies to ameliorate impaired responses against *T. cruzi* and other adverse effects of PEM/MNM in millions of Central and South American people.

##  Conflict of Interest

The authors declare that there is no commercial or other duality of interest associated with this manuscript.
